# Cerebellopontine Angle Primary Choroid Plexus Carcinoma Present in an Adult: Case Report and Literature Review

**DOI:** 10.7759/cureus.13268

**Published:** 2021-02-10

**Authors:** Andrew J Witten, Stephen K Mendenhall, Logan S DeWitt, Alexander Vortmeyer, Aaron Cohen-Gadol

**Affiliations:** 1 Neurological Surgery, Indiana University School of Medicine, Indianapolis, USA; 2 Department of Neurological Surgery, Indiana University Health, Indianapolis, USA; 3 Department of Pathology and Laboratory Medicine, Indiana University Health, Indianapolis, USA

**Keywords:** primary choroid plexus carcinoma, cerebellopontine angle tumor, adult

## Abstract

Choroid plexus tumors (CPTs) are rare intraventricular neoplasms that primarily occur in children and are rare in adults. Of the CPT subtypes, choroid plexus carcinomas (CPC) are highly aggressive and malignant and of World Health Organization (WHO) Grade III. Dissemination through the cerebrospinal fluid space is the inevitable natural course of the disease.

In this case report, we present a 33-year-old female with a past medical history notable for schizophrenia and bipolar disease who suffered from left-sided acute vision loss and hearing loss. Magnetic resonance imaging (MRI) demonstrated multiple enhancing masses found in the left cerebellopontine angle (CPA), right internal auditory canal, the atrium of the left ventricle, and the left foramen of Monroe. After surgical decompression of the CPA tumor, the permanent final pathology was consistent with CPC.

To our knowledge, this is the first reported case of a primary CPC occurring within the CPA in an adult. The unique presentation and progression of this rare adult-onset CPC provide insight for the diagnosis and treatment of other rare instances of CPTs.

## Introduction

Choroid plexus tumors (CPT) are rare and account for 0.3%-0.6% of all brain tumors [1]. CPTs include choroid plexus papilloma, atypical choroid plexus papilloma, and choroid plexus carcinoma (CPC). Of these subtypes, CPC is the most aggressive and malignant (World Health Organization (WHO) grade-III). CPC is primarily a pediatric disease but presents in about 20% of adults and accounts for 15%-20% of all choroid plexus tumors [2]. CPC is derived from modified ependymal cells and commonly presents with hydrocephalus related to ventricular obstruction. Metastatic lesions disseminate through the cerebrospinal fluid (CSF). In children, they frequently arise from the lateral ventricles and the fourth ventricle in adults. Rarely, they occur in the cerebellopontine angle (CPA), suprasellar region, or even in the sacral canal [3]. To the author's knowledge, we present the first adult case of CPC presenting in the CPA.

Additionally, we conducted a literature review of adult CPC to investigate tumor location, clinical features, symptom duration, surgical outcomes, and adjuvant therapy. A comprehensive search of the literature was performed using Google Scholar and PubMed without date restrictions. During the search, a variety of terms were used in Boolean logic, to include: (1) CPC; (2) adult; (3) CPA; and (4) CPTs. Peer-reviewed studies that focused on CPCs primarily in adult populations were included in the literature review.

## Case presentation

Preoperative course

**Figure 1:**
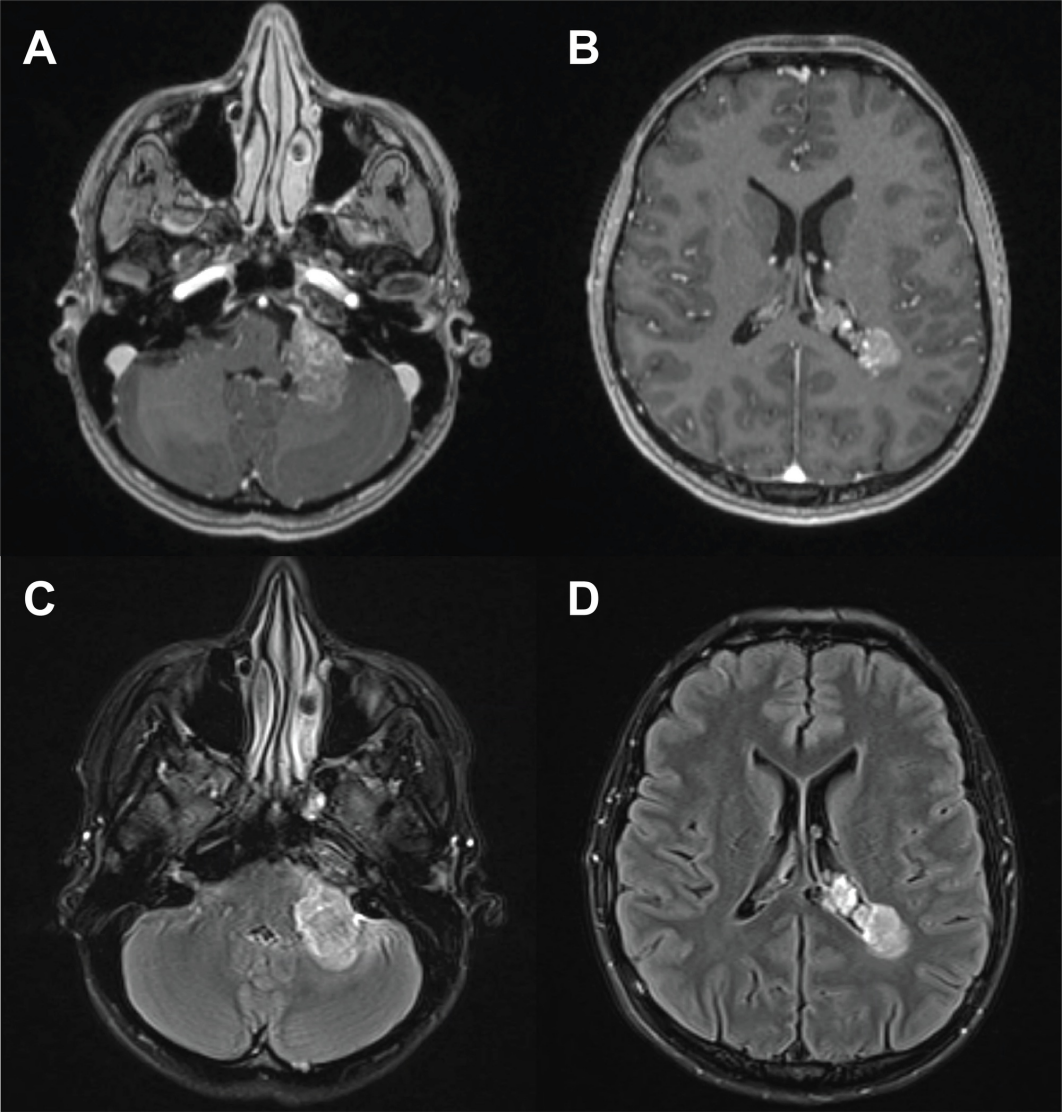
Preoperative brain MRI of the patient (A) Axial view of the left CPA mass located on T1. (B) Axial view of the lesion located in the atrium of the left ventricle on T1. (C) Axial view of the left CPA mass on T2-FLAIR. (D) Axial view of the left ventricle lesion on T2-FLAIR. MRI: magnetic resonance imaging; CPA: cerebellopontine angle; T2-FLAIR: T2-weighted-fluid-attenuated inversion recovery

The patient is a 33-year-old woman who presented to the emergency department with decreased left visual acuity, hearing loss in her left ear, and paresthesia on the left side of her face. Her past medical history was notable for schizophrenia, bipolar disorder, intravenous (IV) drug use, and a 30 pack-year smoking history. On physical exam, she had diminished vision in her left eye, hearing loss in her left ear, and mild aphasia; otherwise, she had a normal neurologic exam. Magnetic resonance imaging (MRI) revealed four lobulated heterogeneous enhancing masses in the left CPA, right internal auditory canal, the atrium of the left ventricle, and the left foramen of Monroe (Figure 1). The largest was in the CPA, measuring 2.1 x 3.5 x 3.1 cm with surrounding vasogenic edema. Computed tomography (CT) of the chest, abdomen, and pelvis and MRI total spine were negative for tumor. She was offered retrosigmoid craniotomy for open biopsy and cranial nerve decompression.

Operative description

**Figure 2:**
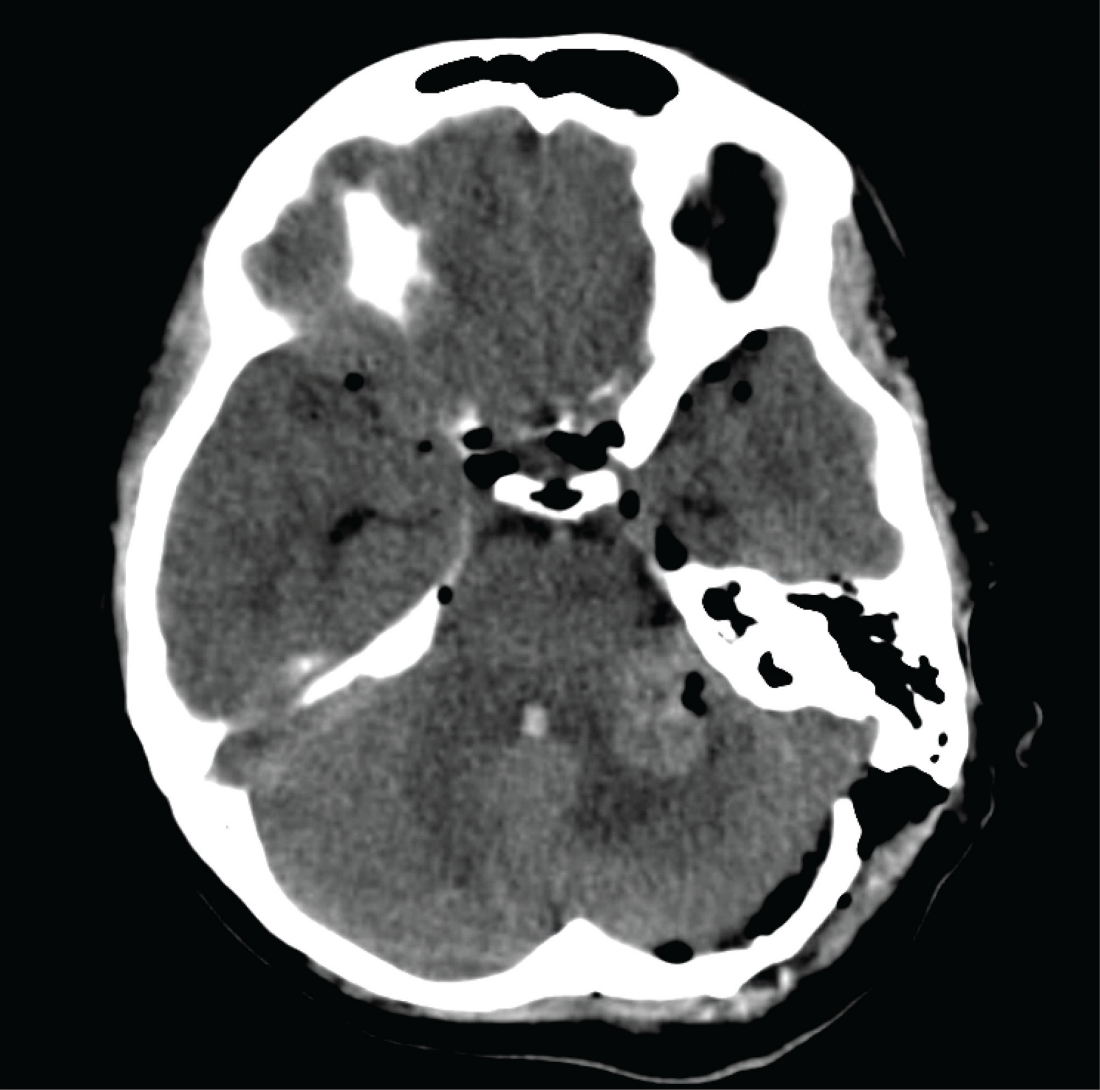
Postoperative brain CT of the patient The left retromastoid entry point is visualized by the defect in the temporal bone. Subtotal resection of the tumor shows decompression of the CPA. CT: computed tomography; CPA: cerebellopontine angle

The patient was positioned in the lateral position with the left side up. Cranial nerves 7-12 were monitored. An intraoperative lumbar puncture was performed for brain relaxation, and CSF was sent for cytological analysis. A 10 cm curvilinear incision was placed just below an imaginary line between the inion and external auditory canal. A single burr hole was made at the transverse-sigmoid junction, followed by a small retromastoid craniotomy. The craniotomy edges were burred until the edge of the transverse and sigmoid sinuses were visualized. The dura was opened in a C fashion along the transverse sigmoid junction. Using the interoperative microscope, we removed a large section of the tumor and sent it for frozen and permanent pathological specimen. The tumor was debulked utilizing the ultrasonic aspirator while frequently checking the tumor capsule for cranial nerve function using the monopolar Prass probe (Medtronic, Fridley, Minnesota). There was significant tumor adherence to surrounding vessels and cranial nerves. The intraoperative frozen pathology report was consistent with metastatic adenocarcinoma. Given the significant adherence of the tumor to the cerebellum and cranial nerves, we decided to leave the remaining tumor to avoid cranial nerve deficit. The bone flap was replaced at the end of the procedure. Routine postoperative CT was performed (Figure 2).

Postoperative course

The patient’s preoperative left visual acuity and facial paresthesia resolved. Her hearing loss remained after surgery. She was otherwise neurologically intact. The patient left against medical advice prior to postoperative MRI and has not reported for postoperative or oncologic follow-up.

Pathological findings

**Figure 3:**
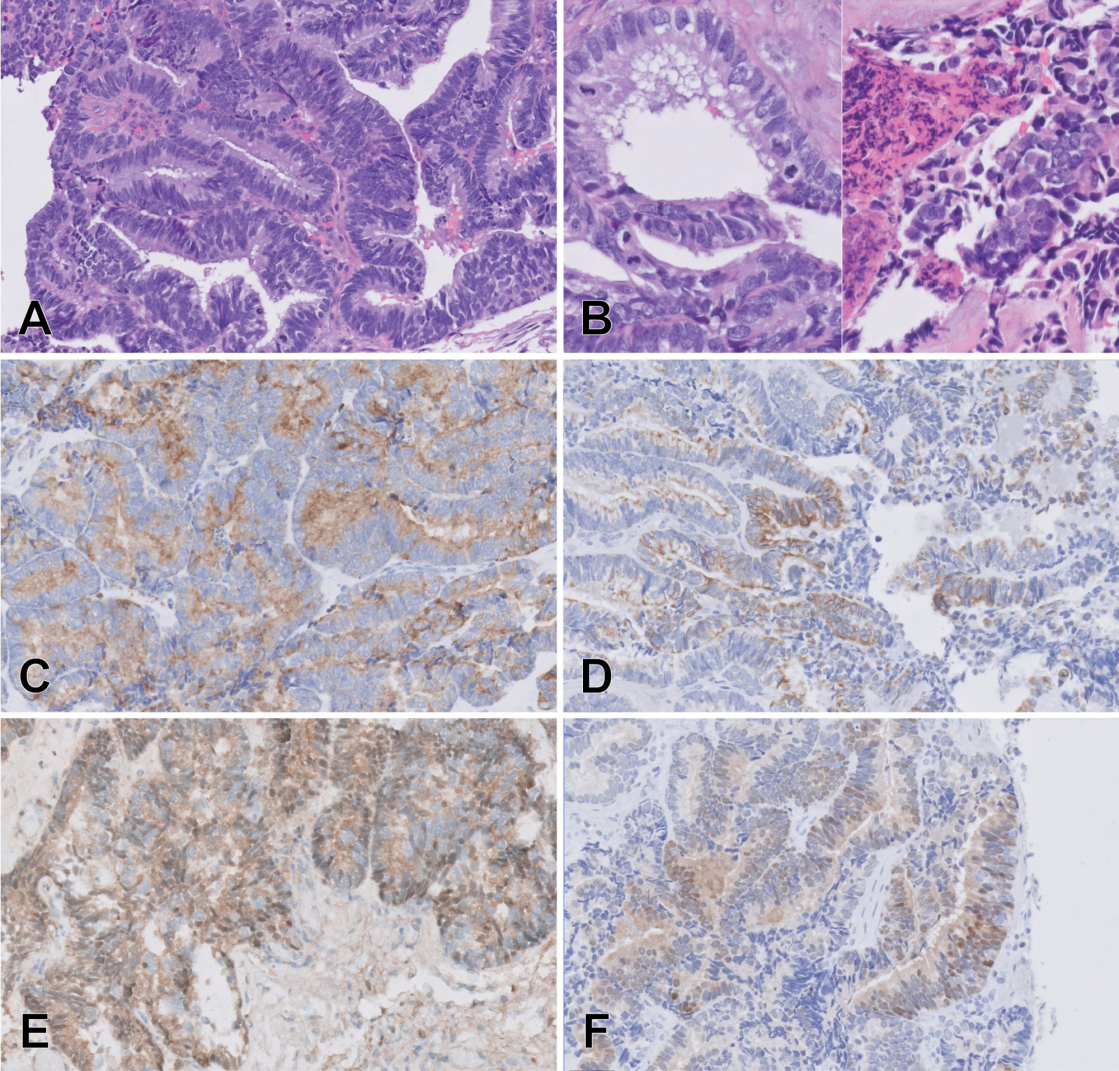
Photomicrographs of immunohistochemically stained choroid plexus carcinoma surgical specimens (A) H&E, 20x. (B) Increased mitotic activity and necrosis, H&E, 40x. By immunohistochemistry, the tumor cells are positive for (C) CK7, 20x; (D) S100, 20x; (E) transthyretin, 20x; and (F) synaptophysin, 20x.

The tumor consisted of pleomorphic epithelial cells, frequently with vacuolated cytoplasm, arranged in a well-defined papillary pattern (Figures 3A-3F). The tumor cells were positive for AE1/AE3, CK7, prealbumin (transthyretin), S100, synaptophysin, and vimentin. They were negative for CEA, CK20, EMA, ER, GATA3, GFAP, Napsin A, p16, PAX8, TTF1, and WT-1. Rare scattered cells were positive for p53. The Ki67 index was visually assessed as 30%-40%. The special stains and immunohistochemical preparations exclude both ependymoma and adenocarcinoma and were consistent with primary choroid plexus carcinoma, WHO grade III.

## Discussion

Choroid plexus carcinoma is a highly aggressive, malignant tumor that is more common in pediatrics than in adults. To our knowledge, this is the first case report of CPC presenting in the CPA of an adult patient.

CPC stains positive for cytokeratin and has a variable expression for vimentin, S100, transthyretin, and glial fibrillary acidic protein. CPC is typically negative for epithelial membrane antigen. Our patient was positive for CK7, S100, vimentin, and transthyretin while negative for glial fibrillary acidic protein and epithelial membrane antigen. Gross pathology will usually show a friable papillary or cauliflower-like appearance. Papillary ependymoma is a rare variant of ependymoma and often gives rise to confusion with choroid plexus tumors because of topographic, light microscopic, and ultrastructural similarities. Immunohistochemistry for our patient also revealed scattered p53 positivity. Many cases of choroid plexus carcinoma are associated with Li-Fraumeni syndrome, an autosomal dominant disorder involving a mutation in the germ cell line of p53 tumor suppressor genes [4]. Roughly 50%-60% of patients with CPC were observed to have TP53 gene mutations [5]. Her extended neuro-oncology gene panel reported a positive BCL6 corepressor (BCOR) gene mutation. BCOR gene mutations have rarely occurred in high-grade neoplasms but have never been linked directly with a case of CPC [5-6].

CPCs arise from modified ependymal cells surrounding a core of capillaries and loose connective tissue. CPCs are typically intraventricular, however, extraventricular tumors have been reported [7-9]. Ectopic sites are not commonly reported but include the suprasellar region, foramen magnum, and spinal canal in the absence of other intracranial lesions [10]. Ectopic CPC is thought to arise through CSF dissemination [10]. Our literature search found only two occurrences of cerebellopontine angle CPCs; both had an invasion of CPC from the fourth ventricle through the foramen of Luschka into the CPA [11-12]. The most common site for adults is in the fourth ventricle (63%) while in the pediatric group is the lateral ventricle (72%) [10]. Presentation with multiple CPCs is rare, occurring in only 5% of CPC patients [9]. Metastatic lesions have a higher frequency of being supratentorial [10]. Our patient uniquely presented with a primary CPC in the atrium of the left ventricle and ectopic in the CPA without any invasion from the fourth ventricle. Her metastatic lesions were supratentorial in the left foramen of Monroe and the right internal auditory canal. CPC is an aggressive type of brain tumor and should remain on differentials where primary tumors present intraventricularly and in ectopic regions on imaging in adults.

The symptoms of CPC typically relate to progressive obstructive hydrocephalus due to their intraventricular location. Headache, diplopia, nausea, vomiting, and ataxia are the most common presenting symptoms (Table 1). However, when CPC tumors invade into rare locations, such as the CPA, headache and hearing problems start first, followed by gait disturbance, facial nerve involvement, and visual field deficits. Our patient had hearing loss and facial hypesthesia, which is much more common with CPA-based tumors.

**Table 1:** Summary of cases reported as choroid plexus carcinoma FV: fourth ventricle, LV: lateral ventricle, R: right, L: left, GTR: gross total resection; SR: subtotal resection; CT: chemotherapy, RT: radiation therapy

Study	Age/Sex	Symptom Duration	Clinical Features	Location	Craniotomy Location	Surgical Result	Adjuvant Therapy	Outcome
Tham et al [12]	66/F	3 mon	Left facial hemiparesis, dizziness, headache	FV through the foramen of Luschka into the CPA	Suboccipital	Biopsy	None	Expired 2 days after the operation
Izci et al [14]	54/M	~	Headache, visual, gait disturbances	R LV	Transcallosal	GTR	RT	Expired 12 mon after surgery
Osada et al [15]	53/M	5 mon	Gait disturbance, L hemiparesis	R LV	Superior Parietal	SR	RT	No evidence of recurrence 7 mon after the operation
Tena-Suck et al [16]	18/M	12 mon	Headache, nausea, vomiting, visual disturbance, diplopia, ataxic gait, L-sided hemiparesis	FV	Suboccipital	GTR	None	No evidence of recurrence 2 years after the operation
Lozier et al [7]	68/F	8 mon	Mild expressive aphasia, confusion, difficulty concentrating	L anterior temporal (extraventricular)	Frontotemporal	GTR	CT and RT	No evidence of recurrence 4 years after the operation
Kishore et al [4]	24/M	2 mon	Headache, nausea, vomiting	R LV (temporal horn)	Parietal	GTR	RT	~
Yip et al [17]	21/M	1 mon	Headache	L LV (trigone and occipital horn)	Occipitoparietal, posterior interhemispheric precuneus	GTR	CT and RT	No evidence of recurrence 2 years after the operation
Ozdogan et al [18]	73/M	~	Headache	FV	Transcortical	GTR	None	~
Bohara et al [3]	60/M	~	Headache	R LV (trigone)	Transcortical, middle temporal gyrus	SR	CT and RT	Expired 13 mon after the operation
Guo et al [8]	59/M	1 mon	Speech delay	L temporoparietal (extraventricular)	Temporoparietal	GTR	CT and RT	Recurred 6 mon after resection
Pellerino et al [19]	50/M	~	Dizziness, progressive gait disturbance	FV	~	GTR	CT and RT	Recurred 8 years after resection
Zhu et al [20]	34/F	~	Headache, double vision, nausea	L LV	~	GTR	RT	~
Azhani et al [10]	39/M	2 weeks	Headache, L hemiparesis	R temporoparietal	Trans-sylvian	SR	~	Expired 2 weeks after surgery
Kim et al [9]	49/F	1 week	Visual disturbance, generalized tonic-clonic seizure	Temporoparietal (extraventricular)	Transcortical via parieto-occipital junction	GTR	CT	No evidence of recurrence 10 mon after the operation
	40/M	~	Dizziness, nausea, and headache	FV, tectum	~	SR	CT and RT	Expired 11 mon after the operation
This study	33/F	4 mon	Headache, L-sided hearing loss, L paresthesias, L vision loss	L CPA, R IAC, L LV (atrium), and L foramen of Monroe	Retromastoid	SR	TBD	~

Adjuvant therapy for CPC is controversial. In 2009, Wrede published a meta-analysis showing chemotherapy improved the survival of patients with resected CPC. Patients with CPC receiving combined radiation and chemotherapy had the best two-year survival (63%), followed by those with chemotherapy alone (45%), radiotherapy alone (32%), and those without further therapy (15%) [13]. Various agents have been shown to reduce the size of CPCs after resection such as cisplatin, etoposide, cyclophosphamide, procarbazine, carboplatin, methotrexate, doxorubicin, and vincristine [13]. With its rarity, adjuvant chemotherapy after resection remains controversial as the standard of care for patients with CPC [2]. Although the mainstay of treatment of CPC tumors is attempting a gross total resection, post-resection chemotherapy and radiation should be considered as a rational treatment option to improve outcomes.

## Conclusions

Choroid plexus carcinoma presenting in the CPA is extremely rare. Gross total surgical resection was limited due to cranial nerve invasion. This is the first reported case of a primary CPC occurring within the CPA in an adult. The unique presentation and progression of this rare adult-onset CPC provide insight for the diagnosis and treatment of other rare instances of CPTs.
